# The Significance of Platelet Indices in the Evaluation of Thrombocytopenia

**DOI:** 10.7759/cureus.65756

**Published:** 2024-07-30

**Authors:** Subbiah Senthil Nathan, Priyadarshini Varadaraj, Gunasekaran Nallusamy, Keesari Sai Sandeep Reddy

**Affiliations:** 1 Internal Medicine, Saveetha Medical College and Hospital, Saveetha Institute of Medical and Technical Sciences, Saveetha University, Chennai, IND

**Keywords:** hematology, hyperdestructive thrombocytopenia, hypoproductive thrombocytopenia, plateletcrit, platelet function disorders, thrombocytopenia, platelet indices

## Abstract

Introduction: Thrombocytopenia, characterized by a low platelet count, poses a risk of abnormal bleeding. Traditional diagnostic methods focus on platelet count alone, but emerging evidence suggests that platelet indices like mean platelet volume (MPV) and platelet distribution width (PDW) could provide valuable insights. This study aims to investigate the role of platelet indices in thrombocytopenia assessment, exploring their potential as additional diagnostic and prognostic markers.

Methodology: Over a five-month period, this prospective study enrolled 80 adult patients with thrombocytopenia. Platelet indices were measured using an automated blood cell analyzer (SYSMEX XN 1000; Sysmex Corporation, Kobe, Japan), and statistical analyses were performed using the Statistical Package for the Social Sciences (IBM SPSS Statistics for Windows, IBM Corp., Version 23.0, Armonk, NY).

Results: The study participants showed significant age differences between hypoproductive and hyperproductive thrombocytopenia groups but no significant gender-based disparities. While platelet count and plateletcrit (PCT) didn't differ significantly between groups, individuals with hyperdestructive thrombocytopenia had higher MPV values. Platelet indices varied across clinical conditions, highlighting their potential diagnostic and prognostic value.

Conclusion: Platelet indices like MPV, along with platelet count and PCT, offer insights into thrombocytopenia causes and prognosis. Further research is needed to confirm these findings, but integrating platelet indices into clinical practice could inform treatment decisions and reduce unnecessary procedures like bone marrow biopsies and imaging studies.

## Introduction

Thrombocytopenia, defined as a platelet count below 150,000 cells per cubic millimeter, stands as the leading cause of abnormal bleeding. Clinical manifestations range from minor bruising to life-threatening hemorrhage, correlating with the severity of thrombocytopenia. Notably, literature documents a surge in spontaneous mucosal bleeding, encompassing intracranial and gastric hemorrhage, in cases with counts below 10,000 cells per cubic millimeter [1]. The introduction of various indices into automated hematology analyzers has revolutionized the comprehension of hematological disorders, including those affecting platelets. Platelet parameters such as mean platelet volume (MPV), platelet distribution width (PDW), and platelet large cell ratio (P-LCR) emerge as promising new biomarkers across several diseases such as immune thrombocytopenia, myeloproliferative disorders, systemic lupus erythematosus, acute coronary syndrome, etc. owing to their accessibility and cost-effective measurement methods [2].

The evaluation of thrombocytopenia is crucial in clinical practice as it often serves as an indicator of underlying pathologies and guides therapeutic decisions. Traditional diagnostic approaches primarily rely on assessing platelet count alone, but emerging evidence suggests that platelet indices, including plateletcrit (PCT), PDW, MPV, and P-LCR offer valuable insights into the pathophysiology and prognosis of thrombocytopenia [3].

The evaluation of thrombocytopenia poses diagnostic challenges due to its heterogeneous nature, ranging from benign transient causes to life-threatening conditions such as immune thrombocytopenic purpura (ITP) and hematologic malignancies. Traditional diagnostic algorithms primarily rely on platelet count thresholds to differentiate between various etiologies and guide clinical management. However, platelet indices, which reflect the size, distribution, and functionality of platelets, offer additional insights into the underlying pathophysiology of thrombocytopenia [4]. For example, increased MPV is associated with platelet activation and turnover, while alterations in PDW may indicate abnormalities in platelet production or destruction. Integrating platelet indices into the diagnostic workup of thrombocytopenia has the potential to enhance diagnostic accuracy, risk stratification, and prognostication, ultimately improving clinical outcomes [5].

Current diagnostic challenges in thrombocytopenia revolve around the limitations of conventional methods that predominantly focus on platelet count alone. While platelet count serves as a fundamental parameter in diagnosing thrombocytopenia, it may not fully capture the complexity of the condition. For instance, patients with mild thrombocytopenia may exhibit bleeding tendencies disproportionate to their platelet count, while others with severe thrombocytopenia may remain asymptomatic. Platelet indices, such as MPV, PDW, P-LCR, and immature platelet fraction (IPF), offer valuable insights as MPV indicates platelet activation and is associated with thrombotic risk and various diseases. PDW reflects platelet size variability, indicating active platelet production and destruction. P-LCR measures the proportion of large, reactive platelets, useful for assessing thrombotic risk. IPF indicates bone marrow activity and helps differentiate causes of thrombocytopenia [3]. Incorporating these parameters into the diagnostic workup of thrombocytopenia can provide a more comprehensive assessment of platelet status, aiding in the differentiation of underlying etiologies and risk stratification. Therefore, there is a pressing need to explore the role of platelet indices in conjunction with traditional diagnostic measures to optimize the evaluation and management of thrombocytopenia in clinical practice. This study aims to explore the role of platelet indices in the evaluation of thrombocytopenia, shedding light on their potential utility as adjunctive diagnostic and prognostic markers.

## Materials and methods

This prospective observational study was conducted in the Department of General Medicine at Saveetha Medical College Hospital. Patients were recruited from both outpatient and inpatient departments using convenience sampling methods. Baseline data, including demographic details, medical history, and clinical parameters, were collected, along with regular follow-up data on clinical outcomes and adverse events. Data were recorded using structured questionnaires and electronic medical records, anonymized, and securely stored. Key variables included demographic factors, clinical parameters, and lifestyle factors, with primary and secondary outcomes defined by specific criteria, such as symptom reduction and quality of life improvements. Written informed consent was obtained from all participants after explaining the study's purpose, procedures, risks, and benefits, with the study receiving approval from the Institutional Ethics Committee of Saveetha Medical College Hospital. Patients were divided into two groups: hypoproductive thrombocytopenia and hyperproductive thrombocytopenia.

Hypoproductive thrombocytopenia is a condition that occurs when the bone marrow produces an insufficient number of platelets in which the MPV is low. Causes include bone marrow disorders like aplastic anemia, myelodysplastic syndromes, leukemia, and the effects of chemotherapy or radiation therapy. The result is a reduced platelet count, leading to an increased risk of bleeding.

Hyperproductive thrombocytopenia refers to situations where platelet destruction or consumption outpaces the production despite the bone marrow's increased effort to produce more platelets in which the MPV is high and increased PDW. Causes can include conditions like immune thrombocytopenia (ITP), disseminated intravascular coagulation (DIC), or thrombotic thrombocytopenic purpura (TTP). Although platelet production is elevated, the overall platelet count remains low due to the rapid destruction or utilization of platelets. The comparison of hypoproductive and hyperproductive thrombocytopenia in this study is crucial for accurate diagnosis, understanding different pathophysiological mechanisms, tailoring treatment approaches, and improving patient outcomes. This comprehensive approach enhances the overall management and therapeutic strategies for patients with thrombocytopenia. The study was conducted over a span of five months from November 2023 to March 2024.

Inclusion and exclusion criteria

The inclusion criteria for the study were all adult patients of either gender presenting with a platelet count below 150,000 cells, individuals without other underlying conditions known to significantly impact platelet count independently of the thrombocytopenia being studied (e.g., bone marrow failure syndromes), and patients who were clinically stable and able to provide a reliable medical history.

The exclusion criteria for the study were patients under treatment with antiplatelet medications or any other drugs known to induce thrombocytopenia, pregnant women due to the potential influence of pregnancy on platelet count and other blood parameters, and individuals who have undergone major surgery in the past month, which may affect platelet levels.

Sample size

A total of 80 cases of patients diagnosed with thrombocytopenia were enrolled in the study. For a study with a sample size of 80 patients diagnosed with thrombocytopenia, assuming an effect size (Δ) of 20 units, a significance level (α) of 0.05 (Z-score = 1.96), a power (1-β) of 0.80 (Z-score = 0.84), and a standard deviation (σ) of 45 units from previous studies, the sample size is calculated using the formula, n=(Δ(Zα/2​+Zβ​)σ​)^2^

Procedure

Venous blood samples were drawn into tubes containing di-potassium EDTA (ethylenediamine tetra acetic acid), which is commonly used for routine complete blood count (CBC) analysis. Samples exhibiting a platelet count lower than 150,000 cells/cu.mm were selected for further analysis. Relevant clinical information and available diagnostic results, including serological findings, of patients diagnosed with thrombocytopenia were collected. Standard platelet indices such as platelet count (1.5-4 lakhs/cu.mm), PCT (0.22-0.24%), PDW (11 to 22 µm^3^), MPV (8 to 11µm^3^) and P-LCR (18% to 50%) were measured using an automated blood cell analyzer (SYSMEX XN 1000; Sysmex Corporation, Kobe, Japan) and subsequently analyzed. However, reference ranges can vary slightly depending on the laboratory and the population being tested. All these disease categories were based on the International Classification of Diseases, Tenth Revision (ICD-10) category.

Statistical analysis

The statistical analysis for this study involves both descriptive and inferential methods to assess the relationship between platelet indices and thrombocytopenia. Descriptive statistics were provided with summary measures such as means, standard deviations, and frequency distributions of platelet count, PCT, PDW, and MPV in the study population. Statistical analyses were performed using Statistical Package for the Social Sciences (IBM SPSS Statistics for Windows, IBM Corp., Version 23.0, Armonk, NY) with a significance level of p-value < 0.05.

## Results

Table 1 presents the baseline characteristics of the study participants, categorized into two groups based on the etiology of thrombocytopenia: hypoproductive thrombocytopenia (n=25) and hyperproductive thrombocytopenia (n=55). The mean age of participants with hypoproductive thrombocytopenia is 52.66 years with a standard deviation of 8.84, whereas participants with hyperproductive thrombocytopenia have a higher mean age of 58.5 years with a standard deviation of 8.18. The p-value is 0.005 in age between the two groups. In the hypoproductive thrombocytopenia group, 16 participants (62.4%) are male, and nine participants (37.6%) are female. Similarly, in the hyperproductive thrombocytopenia group, 37 participants (66.4%) are male, and 18 participants (33.6%) are female. The p-value of 0.727 suggests that there is no statistically significant difference in gender distribution between the two groups.

**Table 1:** Baseline characteristics of study participants

Parameters	Hypoproductive thrombocytopenia n=25 (%)	Hyperproductive thrombocytopenia n=55 (%)	p-value
Age in years (mean ± SD)	52.66 ± 8.84	58.5 ± 8.18	0.005
Gender			0.727
Male	16 (62.4)	37 (66.4)	
Female	9 (37.6)	18 (33.6)	

**Figure 1:**
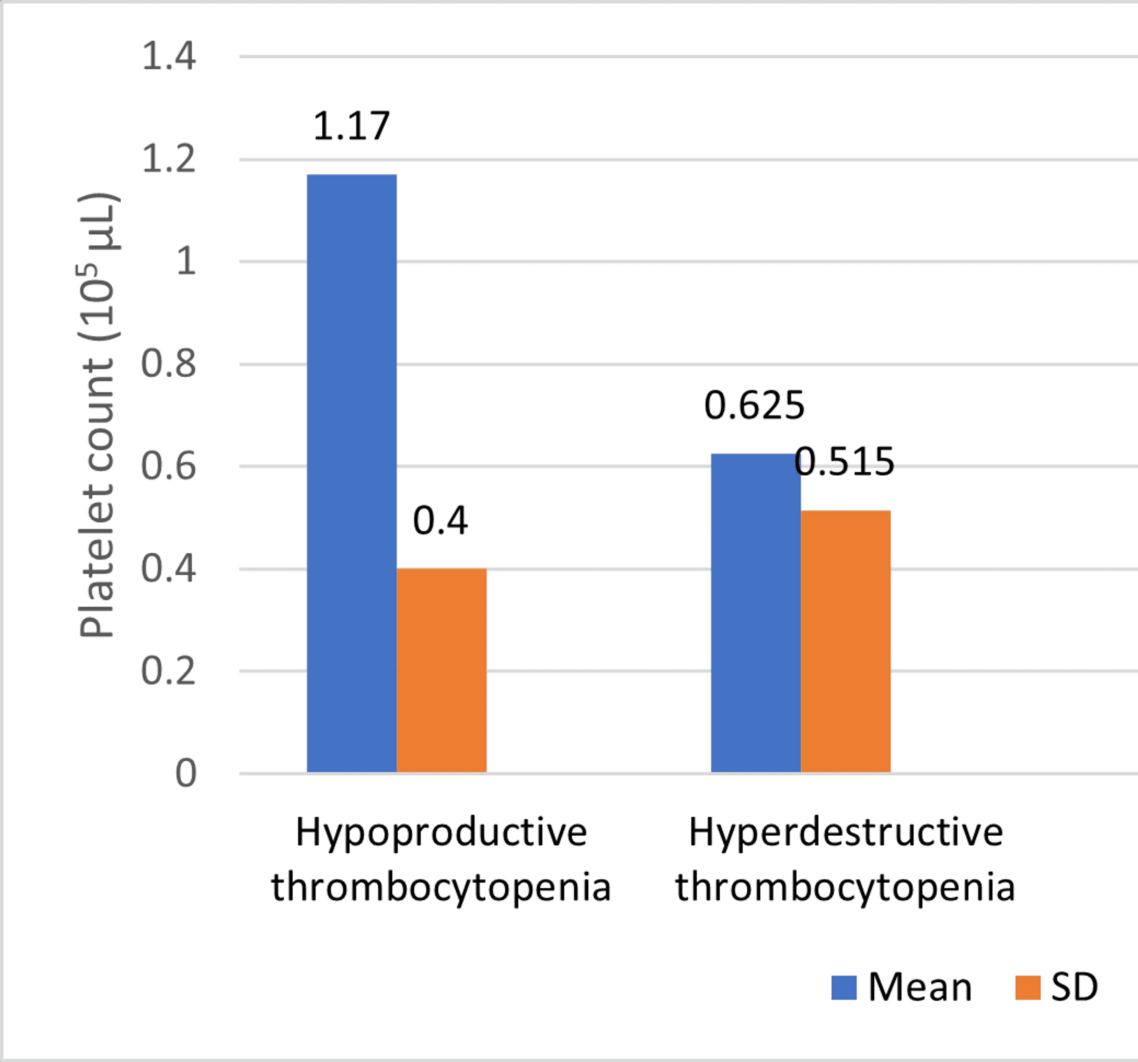
Comparison of platelet count between hypoproductive thrombocytopenia and hyperdestructive thrombocytopenia

**Figure 2:**
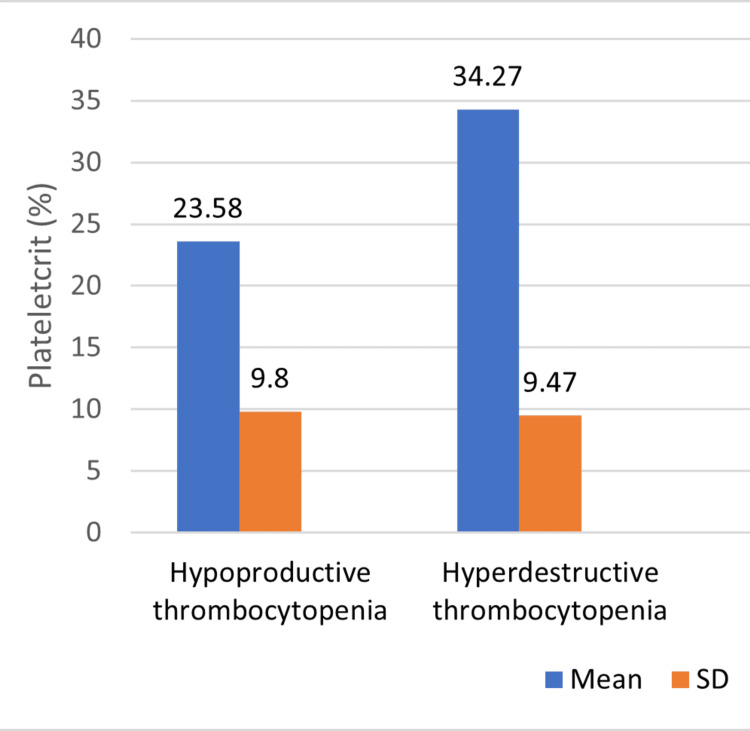
Comparison of platetetcrit (PCT) between hypoproductive thrombocytopenia and hyperdestructive thrombocytopenia

Figures 1-2 compare platelet count and PCT between individuals with hypoproductive thrombocytopenia and hyperdestructive thrombocytopenia. In individuals with hypoproductive thrombocytopenia, the mean platelet count is 1.17 x 10^5 cells/µL with a standard deviation of 0.40. Conversely, individuals with hyperdestructive thrombocytopenia have a lower mean platelet count of 0.625 x 10^5 cells/µL with a higher standard deviation of 0.515. The p-value is 0.09, for PCT, individuals with hypoproductive thrombocytopenia have a mean value of 23.58% with a standard deviation of 9.80, while those with hyperdestructive thrombocytopenia have a higher mean value of 34.27% with a similar standard deviation of 9.47. The p-value of 0.127 suggests that this difference is not statistically significant, again failing to reject the null hypothesis of no difference in PCT between the two groups.

**Figure 3:**
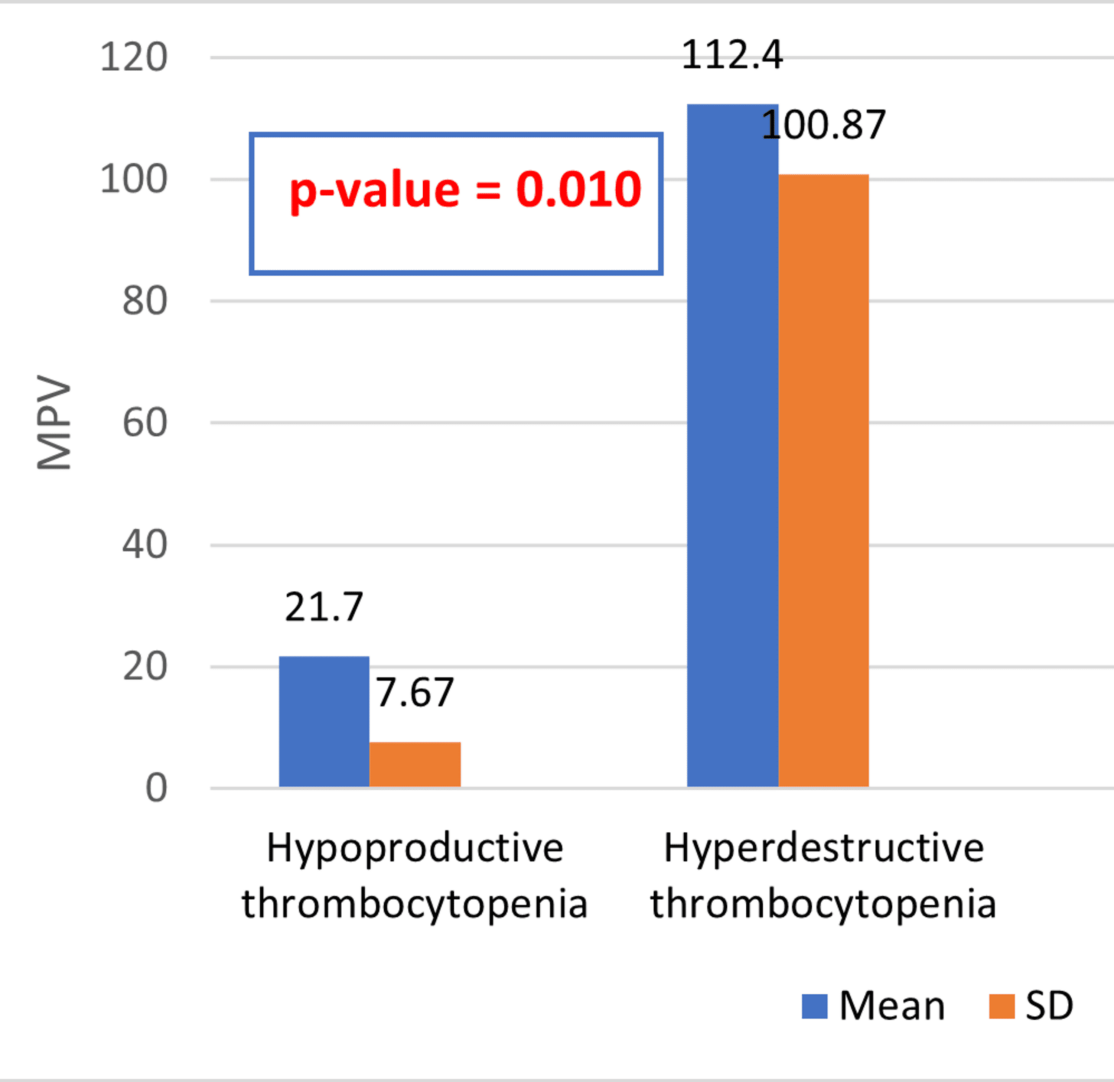
Comparison of mean platelet volume (MVP) between hypoproductive thrombocytopenia and hyperdestructive thrombocytopenia

Figure 3 compares MPV between individuals with hypoproductive thrombocytopenia and hyperdestructive thrombocytopenia. In individuals with hypoproductive thrombocytopenia, the mean MPV is 21.7 ± 7.67 femtoliters (fl), while in those with hyperdestructive thrombocytopenia, the mean MPV is substantially higher at 112.40 ± 100.87 fl. The association between PCT and MPV is expected because PCT is directly influenced by both platelet count and MPV. In thrombocytopenia, where platelet count is reduced, even small changes in MPV can significantly affect PCT values. Higher MPV values, as observed in hyperdestructive thrombocytopenia, contribute to higher PCT values when the platelet count is low, indicating larger, possibly younger platelets compensating for increased platelet destruction. Additionally, the p-value is reported as 0.010.

**Figure 4:**
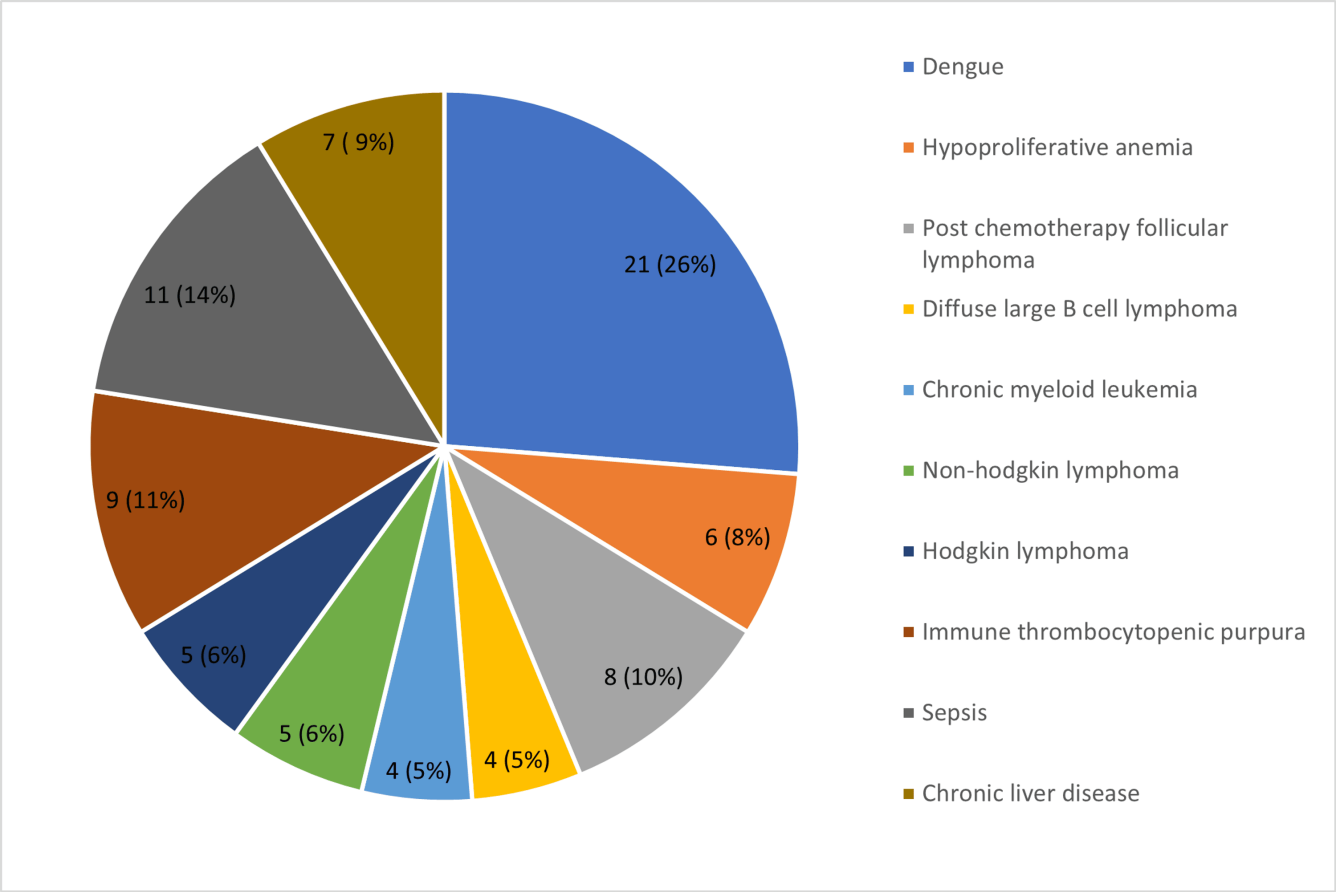
Clinical conditions in the study participants

Figure 4 describes the various clinical conditions of the study participants.

Table 2 presents the distribution of platelet indices under various clinical conditions, offering insights into the characteristics of thrombocytopenia across different disease categories. Dengue fever, characterized by a platelet count of 0.61 ± 0.2 x 10^3 µL, exhibits elevated PCT (39.5 ± 5%) and MPV (11.8 ± 1 fl), indicating platelet activation. In contrast, hypo proliferative anemia is associated with a slightly higher platelet count (0.805 ± 0.15 x 10^3 µL) but lower PCT (12.3 ± 2%) and MPV (10.6 ± 1.5 fl), suggestive of impaired platelet production. Post-chemotherapy follicular lymphoma presents with a markedly reduced platelet count (0.08 ± 0.05 x 10^3 µL) and moderate PCT (20.1 ± 3%), accompanied by normal MPV (11.2 ± 2 fl). Distinct platelet indices patterns are observed across other diseases, reflecting variations in platelet physiology and disease pathogenesis.

**Table 2:** Distribution of platelet indices under various clinical conditions PDW: platelet distribution width; P-LCR: platelet large cell ratio

Disease categories	Platelet count (10^5 µL)	Plateletcrit (%)	Mean platelet volume (MPV)	PDW	P-LCR
Dengue	0.61 ± 0.2	39.5 ± 5	11.8 ± 1	17.7 ± 3	21.2 ± 4
Hypo proliferative anemia	0.805 ± 0.15	12.3 ± 2	10.6 ± 1.5	15.2 ± 2.5	22.1 ± 3
Diffuse large B cell lymphoma	1.84 ± 0.3	32.1 ± 4	8.8 ± 1	9.67 ± 2	30.6 ± 5
Chronic myeloid leukemia	1.2 ± 0.25	35 ± 4	8.61 ± 1.5	9.2 ± 2	32.2 ± 5
Non-Hodgkin lymphoma	0.98 ± 0.2	26.8 ± 3	9.21 ± 1.5	9.5 ± 2	35.4 ± 4
Hodgkin lymphoma	1.4 ± 0.3	23 ± 3	10.9 ± 1.5	8.9 ± 2	34.8 ± 4
Immune thrombocytopenic purpura	1.2 ± 0.25	38 ± 5	11.24 ± 1.5	15.8 ± 2.5	26.2 ± 3
Sepsis	0.9 ± 0.2	28 ± 4	12.1 ± 1	11.6 ± 2	18.6 ± 3
Chronic liver disease	0.82 ± 0.2	22 ± 3	11.62 ± 1.5	12.8 ± 2	22.6 ± 3

## Discussion

The results presented in the study provide insights into the baseline characteristics of the study participants, stratified based on the etiology of thrombocytopenia. Significant differences were observed in the mean age between individuals with hypoproductive thrombocytopenia (mean age = 52.66 years) and those with hyperproductive thrombocytopenia (mean age = 58.5 years), with the latter group exhibiting a higher mean age. This age-related difference could influence disease severity or treatment response for several reasons. Firstly, aging is associated with changes in the bone marrow that can affect hematopoietic function, potentially leading to different types of thrombocytopenia based on age. Secondly, older individuals often have a higher burden of comorbidities such as chronic inflammatory diseases, malignancies, and myeloproliferative disorders, which can contribute to hyperproductive thrombocytopenia. Thirdly, chronic diseases and their treatments, more prevalent in older adults, can impact bone marrow function and platelet production. Finally, immunosenescence, the aging of the immune system, may alter immune responses and contribute to the development of hematological disorders, including hyperproductive thrombocytopenia [4]. These factors highlight the complex interplay between aging and thrombocytopenia, necessitating age-specific considerations in diagnosis and treatment. However, no significant gender-based differences were detected between the two groups. These findings suggest that age may play a role in thrombocytopenia etiology, potentially influencing disease severity or treatment response.

Dengue is characterized by a significantly low platelet count with high PCT, high MPV, and high PDW, indicative of platelet destruction and a compensatory increase in young, large platelets. Hypoproliferative anemia shows a moderately low platelet count and very low PCT, with normal MPV and PDW, reflecting bone marrow failure. Post-chemotherapy follicular lymphoma presents with an extremely low platelet count and high PCT, reflecting the impact of chemotherapy on bone marrow. Diffuse large B cell lymphoma displays an elevated platelet count, high PCT, low MPV, low PDW, and high P-LCR, indicating increased platelet production but with smaller platelets. Chronic myeloid leukemia exhibits a moderately elevated platelet count with high PCT, low MPV, and low PDW, reflecting increased production of smaller platelets. Non-Hodgkin lymphoma shows a normal to slightly elevated platelet count with high PCT and high P-LCR, indicating a robust production of larger platelets [5]. Hodgkin lymphoma is marked by an elevated platelet count and moderate PCT, with normal MPV and low PDW. ITP features a normal to elevated platelet count with high PCT and high PDW, indicative of increased platelet destruction and compensatory production. Sepsis presents with a low platelet count, moderate PCT, high MPV, and moderate PDW, reflecting platelet consumption in response to infection. Sepsis is a life-threatening condition that happens when the body’s immune system has an extreme response to an infection, causing organ dysfunction. Chronic liver disease exhibits a low to normal platelet count with low PCT, high MPV, and moderate PDW due to the liver's role in platelet production and destruction. By examining these parameters together, clinicians can better understand the underlying pathology and make more accurate differential diagnoses.

Figures 1-2 depict the comparison of platelet count and PCT between individuals with hypoproductive and hyperdestructive thrombocytopenia. While no statistically significant differences were observed in platelet count or PCT between the two groups, these figures provide valuable insights into the variability of these parameters across different etiologies of thrombocytopenia as they show the variability of these parameters across different etiologies of thrombocytopenia. Figure 3 further explores the differences in MPV between individuals with hypoproductive and hyperdestructive thrombocytopenia. A statistically significant difference was observed in MPV between the two groups, with individuals in the hyperdestructive thrombocytopenia group exhibiting substantially higher MPV values compared to those with hypoproductive thrombocytopenia. This finding suggests potential differences in platelet physiology or function between the two groups, which may have clinical implications for disease management and prognosis.

Additionally, this study also provides valuable insights into the distribution of platelet indices across various clinical conditions associated with thrombocytopenia. The observed variability in platelet count, PCT, MPV, PDW, and P-LCR among different disease categories underscores the heterogeneity of thrombocytopenia etiologies and highlights the potential utility of platelet indices as diagnostic or prognostic markers in clinical practice because the variability observed in platelet indices such as platelet count, PCT, MPV, PDW, and P-LCR across different disease categories underscores the heterogeneous nature of thrombocytopenia etiologies. Thrombocytopenia can arise from diverse mechanisms including decreased production, increased destruction, or sequestration of platelets, each contributing differently to the profile of platelet indices. For instance, conditions like ITP often exhibit low platelet count alongside high PCT and PDW, reflecting accelerated platelet turnover and compensatory production. In contrast, chronic liver disease may present with normal to low platelet count, low PCT, and altered MPV and PDW due to impaired hepatic function affecting platelet production and turnover [6]. The distinct patterns in these indices not only aid in diagnosing specific thrombocytopenic disorders but also serve as prognostic markers by indicating disease severity and guiding therapeutic decisions. Monitoring changes in platelet indices over time can offer insights into treatment response and disease progression, thereby enhancing clinical management strategies tailored to individual patient needs.

In our study encompassing 80 cases of thrombocytopenia, patients' ages ranged from 18 to 72 years, with infections (38%) representing the most prevalent cause. Consistent with previous research by Yalavarthi et al. [7] and Mala et al. [8], infections emerged as the primary etiological factor. Dengue fever emerged as the predominant cause, constituting 32% of cases, albeit slightly lower than reported in other studies. The variation in disease prevalence may be attributed to demographic disparities and infection endemicity. ITP accounted for 8.6% of cases in our study. Similar findings were reported by Bessman et al. [9], Kaito et al. [10], Ntaios et al. [11], and Elsewefy et al. [12]. Furthermore, studies by Norrasethada et al [13] and Numbenjapon et al. [14], proposed specific MPV cutoff values for distinguishing between hyperdestructive and hypoproductive groups, underscoring the diagnostic potential of these indices.

In sepsis cases, which accounted for 12% of total cases, the mean MPV was 12.1. Gao et al. [15] highlighted the prognostic value of MPV in septic shock, suggesting that elevated MPV, along with PDW and P-LCR, may indicate a poorer prognosis. They recommended MPV as a potential predictor of mortality, second only to serum lactate dehydrogenase levels. Our study revealed significantly higher MPV, PDW, and P-LCR among patients with hyperdestructive thrombocytopenia compared to the hypo proliferative group. Afsar et al. [16] also noted increased MPV values in dengue cases, while Sharma and Yadav [17] found no significant correlation between MPV and severity or treatment outcomes in dengue cases, indicating varied findings regarding MPV's role in dengue.

In comparing our findings with similar studies, it's essential to consider the methodologies employed, patient demographics, and the specific parameters investigated. While our study focuses on exploring platelet indices and their associations with clinical parameters in thrombocytopenia, similar investigations have examined related aspects of platelet function and disease outcomes [18,19]. Fewer investigations have been conducted to assess the effectiveness of platelet indices in distinguishing between hypo proliferative and hyperdestructive thrombocytopenia. MPV serves as one such parameter, providing an estimate of platelet size and indirectly reflecting their activity [20]. In cases of hyperdestructive thrombocytopenia, there is often a compensatory increase in platelet production, accompanied by the release of immature platelet forms into the bloodstream. As immature platelets tend to be larger than mature ones, the MPV measured by automated blood analyzers is usually higher, particularly in the presence of bone marrow abnormalities [21].

The normal MPV observed in our study can be attributed to several factors related to the analytical methods used and the clinical context of our study population. Automated blood analyzers often standardize MPV measurements to account for variations in platelet size, ensuring consistency in reporting across different samples. This normalization process helps mitigate the influence of factors such as platelet activation or the presence of immature platelets, which typically contribute to higher MPV values in the context of bone marrow abnormalities. Additionally, our study may have sampled from a population where the impact of these abnormalities on MPV was not pronounced or was balanced by other clinical factors. PDW, akin to red cell distribution width, signifies the degree of cellular heterogeneity. Research on MPV and PDW has demonstrated promising sensitivity and specificity in diagnosing thrombocytopenia [22] which showed the mean platelet count in the hypoproduction group is 75.9 ± 36.4 and in the hyperdestruction group is 79.6 ± 36.3 with a p-value of 0.64. The mean MPV in the hypoproduction group is 10.17 ± 1.3 and in the hyperdestruction group is 12.3 ± 0.9 with a significant p-value of 0.05. The mean PDW in the hypoproduction group is 19.7 ± 5.4 and in the hyperdestruction group is 19.3 ± 4.2 with a p-value of 0.7. The mean PCT in the hypoproduction group is 0.06 ± 0.03 and in the hyperdestruction group is 0.08 ± 0.1 with a p-value of 0.2. MPV may provide useful information in discriminating the hypoproductive and hyperdestructive thrombocytopenia. Interpretation of platelet indices can help thrombocytopenic patients in the initial management and can avoid invasive investigations. Platelet activation, which prompts an increase in both the number and size of platelets, can influence the estimated PDW values [23].

Overall, these results contribute to our understanding of the demographic and clinical factors associated with thrombocytopenia and underscore the importance of considering platelet indices in disease evaluation and management. Further research is warranted to elucidate the underlying mechanisms driving the observed differences and to validate the clinical utility of platelet indices in thrombocytopenia management.

However, automated systems have inherent limitations, especially in scenarios where platelet indices may not always be recorded. Common challenges include cases of severe thrombocytopenia, conditions involving red cell fragmentation, and EDTA-induced pseudothrombocytopenia and it is understood that the impact of different methodologies and analyzer calibrations on platelet indices measurements emphasizes the need for standardization in this domain.

## Conclusions

Our study suggests that platelet indices, including MPV, platelet count, PCT, and IPFs play a role in discerning the underlying cause of thrombocytopenia. Among these indices, MPV emerges as particularly reliable in distinguishing between different etiologies. However, there were no statistically significant differences observed in platelet count and PCT between the two groups. Overall, platelet indices offer valuable insights that can guide clinical decision-making, potentially averting unnecessary invasive procedures such as bone marrow aspiration, because by using these platelet indices, clinicians can better categorize thrombocytopenia into hypoproductive (low production) or hyperproductive (high destruction) categories and avoid unnecessary platelet transfusions.
